# The Laplacian spectrum of neural networks

**DOI:** 10.3389/fncom.2013.00189

**Published:** 2014-01-13

**Authors:** Siemon C. de Lange, Marcel A. de Reus, Martijn P. van den Heuvel

**Affiliations:** Department of Psychiatry, Brain Center Rudolf Magnus, University Medical Center UtrechtNetherlands

**Keywords:** brain network, connectome, laplacian, eigenvalues, graph spectrum, network classification, normalized laplacian

## Abstract

The brain is a complex network of neural interactions, both at the microscopic and macroscopic level. Graph theory is well suited to examine the global network architecture of these neural networks. Many popular graph metrics, however, encode average properties of individual network elements. Complementing these “conventional” graph metrics, the eigenvalue spectrum of the normalized Laplacian describes a network's structure directly at a systems level, without referring to individual nodes or connections. In this paper, the Laplacian spectra of the macroscopic anatomical neuronal networks of the macaque and cat, and the microscopic network of the Caenorhabditis elegans were examined. Consistent with conventional graph metrics, analysis of the Laplacian spectra revealed an integrative community structure in neural brain networks. Extending previous findings of overlap of network attributes across species, similarity of the Laplacian spectra across the cat, macaque and C. elegans neural networks suggests a certain level of consistency in the overall architecture of the anatomical neural networks of these species. Our results further suggest a specific network class for neural networks, distinct from conceptual small-world and scale-free models as well as several empirical networks.

## Introduction

The brain is a complex system of structurally and functionally interconnected elements. Within this system, healthy brain function depends on a constant interplay between local information processing and efficient global integration of information, facilitated by neural interactions within a large-scale network of structurally interconnected neurons and brain regions; a network known as the connectome (Sporns et al., 2005; Bullmore and Sporns, 2009). Neural interactions occur on multiple scales, ranging from the microscopic level -considering neuronal cells and their synaptic connections- to the macroscopic level -defining neural projections between large-scale brain regions (Sporns et al., 2005). In the past decades, studies examining the topological structure of neural systems, including the neural systems of mammalian species (Scannell et al., 1995; Modha and Singh, 2010) as well as the connectomes of nematode (Varshney et al., 2011) and diptera species (Pavlou and Goodwin, 2013), have been reported to display several network attributes of an efficient communication architecture. Neural systems show high levels of local clustering and community structure, together with short communication pathways and central communication hubs, suggested to facilitate efficient long-distance communication and global integration of information between subparts of the system (Sporns and Zwi, 2004; Bassett and Bullmore, 2006; Hagmann et al., 2008; Bullmore and Sporns, 2009; Sporns, 2011; van den Heuvel and Sporns, 2011; Harriger et al., 2012; van den Heuvel et al., 2012; Collin et al., 2013).

Neural systems can be mathematically described as a graph *G* = (*V, E*), comprising a collection of nodes *V*, representing neurons or brain regions, and interconnecting edges *E* (Boccaletti et al., 2006; van den Heuvel and Hulshoff Pol, 2010a,b; Sporns, 2011; Bullmore and Sporns, 2012). Commonly explored graph theoretical metrics involve the level of local clustering of nodes, level of degree, level of betweenness or closeness centrality and their level of global and local communication efficiency (Watts and Strogatz, 1998; Newman, 2003; Boccaletti et al., 2006). These “conventional” metrics are primarily focused on mapping the properties of individual nodes, and the examination of overall aspects of network organization, such as the examination of a network's small-world or scale-free features, most often involves a global generalization (for example averaging) of these node metrics to the whole network.

Complementing conventional metrics, an examination of the global structure of neural networks at a systems level may capture qualitative aspects of the network as a whole, including aspects that remain out of sight from conventional measures (Atay et al., 2006; Banerjee and Jost, 2007; Varshney et al., 2011; Banerjee, 2012). In recent years, studies have started to utilize spectral graph theory to examine neural networks (Banerjee and Jost, 2007; Varshney et al., 2011), providing metrics on basis of the eigenvectors of matrices of a network, such as node centrality (Bonacich, 1972, 2007; Page et al., 1999; Lohmann et al., 2010) and community identification methods (Newman, 2006; Fortunato, 2010; Liang et al., 2011; Harriger et al., 2012). While these eigenvectors are still closely related to properties of individual network elements, and are often used as such, the spectrum of the associated eigenvalues contains valuable information related to the overall structure of a graph (Vukadinović et al., 2002; Banerjee and Jost, 2007; McGraw and Menzinger, 2008; Banerjee, 2012), providing new insight in the workings of the brain as a system.

In this paper, we investigate the architecture of neural networks, describing the organization of maps of connections between neural elements (neurons, brain regions), at a systems level by examining the Laplacian eigenvalue spectrum of the connectome data of the macaque, cat and Caenorhabditis elegans. These connectome data are comprehensive descriptions of the anatomical neural networks of the three species, describing the structural connectivity networks of, on the microscopic level, the whole neuronal system (in the C. elegans) or, on the macroscopic level, the networks of long-range white matter projections of the cerebral cortex (in macaque and cat). These three connectome maps are often used in literature (Hilgetag et al., 2000; Sporns and Kötter, 2004; Kaiser, 2011; Zamora-López et al., 2011; de Reus and van den Heuvel, 2013) and in this study, we refer to these neural networks as representatives for a “neural network class.”

The normalized Laplacian matrix, in short referred to as the “Laplacian,” is a transformation of the connectivity matrix of the network, with the Laplacian eigenvalues describing aspects related to global network structure and dynamic interactions among network parts (Chung, 1996; Banerjee, 2012). The spectrum of the normalized Laplacian of undirected networks has the advantage that all eigenvalues are in the domain between 0 and 2, enabling the comparison of networks of different sizes (Banerjee, 2012). Furthermore, it has been noted that similarities between the spectra of networks can be used for the classification of networks (Ipsen and Mikhailov, 2001; Vukadinović et al., 2002; Banerjee and Jost, 2008b; Cetinkaya et al., 2012). Therefore, the spectra of the neural networks are examined in light of providing indications of general organizational characteristics of neural networks across species.

## Materials and methods

### Data

In this study, the Laplacian spectrum of three neural connectivity datasets was examined, describing macroscopic interactions of the macaque and cat brain, and microscopic connectivity of the Caenorhabditis elegans neural system. All connectivity datasets were transformed to undirected and unweighted versions (i.e., all directed edges were transformed to reciprocal binary edges, see also Supplementary Material) to make the analysis of the eigenvalues feasible. A description of the three datasets is included below.

#### Macaque

The connectivity dataset of the macaque was based on tract tracing studies collected in the online Collation of Connectivity data on the Macaque brain (CoCoMac) database [cocomac.g-node.org (Stephan et al., 2001; Kötter, 2004)], analyzed by Modha and Singh (2010), and describes macroscopic white matter projections between cortical regions of the macaque cortex. The connection matrix as examined in this study was a subset of the matrix provided by Modha and Singh (Modha and Singh, 2010) and was taken from the study of Harriger et al. (2012), excluding subcortical areas (thalamus, basal ganglia and brainstem) and including only connected areas (i.e., regions having at least one ingoing and at least one outgoing connection). In all, the included macroscopic connectivity dataset included 242 cortical regions connected by 4090 directed binary connections (Harriger et al., 2012). The density of this matrix, represented by the number of connections divided by the number of possible connections, was 7.0%. In the main analysis of this study, the connectivity data was converted to an unweighted, undirected network, with 6108 connections and 10.5% density. For detailed information on the macroscopic macaque connectome see also (Modha and Singh, 2010; Harriger et al., 2012).

#### Cat

The macroscopic connectivity dataset of the cat cortex was taken from the paper of Scannell et al. (1995), as previously analyzed by e.g., (Hilgetag et al., 2000; Sporns et al., 2004; Zamora-López et al., 2009; de Reus and van den Heuvel, 2013). Similar to the macaque connectome map, this dataset resulted from a literature review, combining information from neuroanatomical literature and tract tracing studies (Scannell et al., 1995). The dataset includes 65 cortical areas connected by 1139 directed weighted connections (all areas having at least one in- and one outgoing connection), giving a density of 27.4%. Three connections strengths were distinguished: weak connections having weight 1, medium strength connections with weight 2 and strong connections with weight 3. For our study, the dataset was converted to a binary, undirected network, including 730 unweighted, undirected connections with a total density of 35.1%.

#### Caenorhabditis elegans

The microscopic connectivity dataset of the Caenorhabditis elegans (C. elegans) was taken from the study (Varshney et al., 2011). The dataset was based on original data from White et al. (1986), and was further improved by Varshney and colleagues, adding new data from serial section electron microscopy reconstructions. The connectivity dataset described 279 neurons, of which 274 were included in this study. Five neurons (IL2DL/R, PLNR, DD06, and PVDR) were excluded as they had not at least one in- and one outgoing connection (Varshney et al., 2011). In total the dataset contained 2956 directed connections by gap-junction and chemical synapses and the density was 3.95%, with connections weighted by the number of junctions between two neurons. For the current analysis, the connectivity matrix was transformed to an undirected, unweighted network having 2253 undirected connections and a density of 6.02%.

#### Model networks

The Laplacian spectra of the neural networks were compared to the spectra of three commonly used model networks. First, the network model by Erdös-Rényi (ER) was considered, in which nodes are linked by randomly placed connections (Erdös and Rényi, 1959). Second, the Watts-Strogatz (WS) model was considered for the examination of the spectra of small-world networks, obtained by randomly rewiring 30% of the connections of a lattice ring network with degree 4 for each node (Watts and Strogatz, 1998). Third, the Barabási-Albert (BA) model was considered to examine the spectra of scale-free networks. BA networks were formed by a stochastic growth process on basis of preferential attachment: starting from a lattice of 4 nodes, a new node was added to the network at every timestep by connecting this node to four already existing nodes with selection probabilities proportional to the degree of the old nodes (Barabási and Albert, 1999). All model networks had 274 nodes and a density close to 6% comparable to the C. elegans network (6.59% density in the WS small-world networks, 6.02% for the ER networks, 5.78% for the BA scale-free networks). Computations showed that the spectra had similar shapes for different numbers of nodes equal to that of the macaque and cat connectome maps. The Laplacian spectra of the model networks were obtained as average over 1000 model networks.

#### Empirical networks

Four empirical networks were examined, describing a food web, an email interchange network, a network of football games and a power grid network. The food web network described the food web of Florida Bay during wet season and consisted of 121 connected living compartments [http://www.cbl.umces.edu/~atlss/ATLSS.html (Ulanowicz et al., 1998)]. After transformation to an undirected, unweighted network, the food web consisted of 1763 undirected connections, with a density of 23.9%. The email interchange network described the email communication at the university of Rovira i Virgili and had 1133 nodes and 5451 undirected unweighted connections, with density 0.85% [http://www.cise.ufl.edu/research/sparse/matrices (Guimerà et al., 2003)]. The American football network described the games between Division IA colleges in 2000 and had 115 nodes and 613 undirected unweighted connections resulting in density 9.35% [http://www.cise.ufl.edu/research/sparse/matrices (Girvan and Newman, 2002)]. The power grid of the Western States of the United States had 4941 nodes, with 6594 undirected and unweighted connections and 0.054% density [http://www.cise.ufl.edu/research/sparse/matrices (Watts and Strogatz, 1998)].

#### Reference networks

The eigenvalues of the Laplacian spectra of the examined networks were compared to average values of 1000 randomized reference networks, which had comparable degree distributions but were randomly wired. These reference networks were obtained by rewiring the connections of the original networks following the Markov random switching algorithm, ensuring preservation of overall network size, network density and degree sequence (Maslov and Sneppen, 2002).

### Laplacian spectrum

#### The normalized Laplacian matrix

In this paper we considered spectra of the normalized Laplacian matrix *L*, which has the advantage that all eigenvalues are in the domain between 0 and 2, enabling the comparison of networks of different sizes (Chung, 1996).

The normalized Laplacian matrix is defined as:

$$L(u,v)=\{\begin{array}{ll} 1 & \text{if }u=v\\ -\frac{1}{\text{deg}u} & \text{if }u\text{ and }v\text{ are connected}\\ 0 & \text{otherwise} \end{array}$$

with *u* and *v* representing two nodes of the network, *L*(*u*,*v*) the edge from node *u* to *v* and deg *u* the (binary) degree of node *u*. Alternatively, the normalized Laplacian can also be expressed in its relation with the adjacency matrix *A* as *L* = *I* − *D*^−1^*A* where *I* is the identity matrix and *D* is a diagonal matrix with *D*(*u*,*u*) = deg *u*. The Laplacian spectrum of the network is then given by the collection of all eigenvalues of *L*; i.e., the collection of all scalars λ for which there exists a non-zero vector *v* (being the associated eigenvector) that satisfies the eigenvalue equation *Lv* = λ*v*.

The normalized Laplacian matrix is unitarily equivalent to the symmetric normalized Laplacian defined by Chung (1996), in which *L* = *I* − *D*^−1/2^*AD*^1/2^, showing that the eigenvalues of both Laplacians are real. Furthermore, the smallest eigenvalue of the normalized Laplacian is always 0 as the constant eigenvector *c*, assigning the same value to each node and thus describing the stationary state of the network, satisfies *Lc* = 0. The multiplicity of the eigenvalues equal to 0 (λ = 0) is equal to the number of connected components, i.e., the number of network parts that are not connected to each other (Chung, 1996). The largest eigenvalue is always equal or smaller than 2, labeling the range of eigenvalues as 0 ≤ λ_1_ ≤… ≤λ_*n*_ ≤ 2 (Chung, 1996).

#### Spectral plots

Spectral plots were obtained from the smoothed eigenvalue distribution Γ (*x*), that consisted of eigenvalue frequencies convolved with a Gaussian kernel,

$$\Gamma (x)=\sum_{i=1}^{n}\frac{1}{\sqrt{2\pi \sigma^{2}}}\exp\left(-\frac{|x-\lambda_{i}{|}^{2}}{2\sigma^{2}}\right)$$

with *n* being the number of eigenvalues and σ being a smoothing factor of 0.015. For the plots, a discrete smoothed spectrum was used in which Γ had steps of 0.001. Furthermore, the distribution was normalized such that the total eigenvalue frequency was one.

#### Spectral distance

The similarity between spectra was quantified using a spectral distance measure, based on the distance measure introduced by Wilson and Zhu (2008), defined as the average Euclidean distance between two spectral plots Γ_1_ and Γ_2_:

$$\begin{array}{l} D(\Gamma_{1},\Gamma_{2})=\frac{1}{n+1}\sum_{i=0}^{n}\underset{j}{\min}\left(\sqrt{{\left(\Gamma_{1}(i)-\Gamma_{2}(j)\right)}^{2}+{\left(i-j\right)}^{2}}\right)\\ \;+\;\frac{1}{n+1}\sum_{j=0}^{n}\underset{j}{\min}\left(\sqrt{{\left(\Gamma_{1}(i)-\Gamma_{2}(j)\right)}^{2}+{\left(i-j\right)}^{2}}\right) \end{array}$$

where Γ (*i*) is the discrete, normalized and smoothed eigenvalue distribution and the number of intervals *n* is 2000. As this distance function is dependent on the scaling of axes, different scales result in different distances. Therefore, this measure of spectral distance is not an invariant distance measure between two networks but only a tool to underpin the visual results quantitatively.

#### Robustness

To investigate the effect of (small) modifications to the neural datasets, reflecting the inclusion of noise to the connectome maps, we examined the spectra of “noise including neural networks.” In the noise including neural networks, a small proportion, respectively 1, 2, and 5% of the edges in the original networks (i.e., macaque, cat and C. elegans) were randomly rewired, such that the degree distributions of the networks were preserved (Maslov and Sneppen, 2002). The upper and lower eigenvalue boundary of 1000 generated noise including networks were plotted and the spectral similarity between the original network and the noise including networks was quantified by computing the average spectral distance between each of the noise including networks and the original network.

## Results

In what follows we describe the Laplacian spectra of three neural networks (i.e., macaque, cat, and C. elegans). We compare these spectra with spectra of commonly used network models and a number of empirical networks and then give a more detailed description of specific properties of the Laplacian spectra of the examined neural networks.

### Laplacian spectrum

#### Neural networks

**Figure 1:**
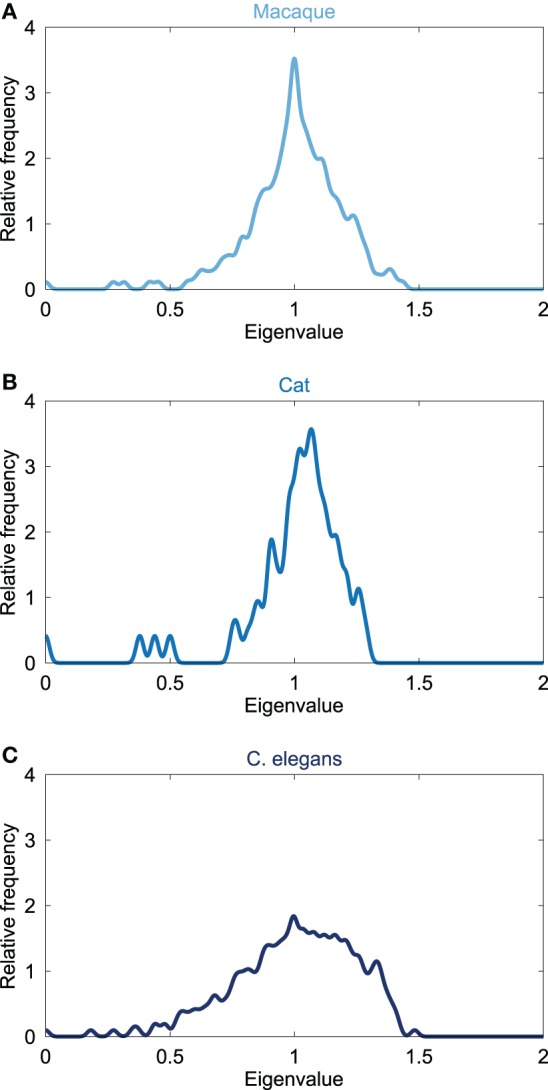
**Laplacian spectra of neural networks**. Figure shows the spectrum of the connectivity networks of the **(A)** macaque (242 cortical regions, 10.5% edge density), **(B)** cat (65 cortical regions, 35.1%), and **(C)** C. elegans (274 neurons, 6.02%). All neural spectra shared three characteristics, being (i) an asymmetric skewed distribution in which the largest eigenvalue was closer to one than the smallest eigenvalue, (ii) a maximum at one, and (iii) a few scattered small eigenvalues. Laplacian spectra showed large overlap across the three neural datasets, suggesting a level of consistency and conservation of overall structure of neural networks.

The Laplacian spectra of the macaque, cat and C. elegans revealed several characteristic properties that were observed for all three networks, see Figure 1. First, all the spectra, from micro- (C. elegans) to macroscale (cat and macaque), showed a left skewed distribution in which the largest eigenvalue was closer to one than the smallest eigenvalue. Second, all distributions showed a peak around one. Third, the first (i.e., smallest) eigenvalues were scattered around a few small peaks at the beginning of the spectra, suggesting similarities in the community structure. The close relation between the Laplacian spectra of these networks suggest shared underlying structural properties of the neuronal systems of the macaque, cat and C. elegans.

#### Comparison of neural networks to network models

**Figure 2:**
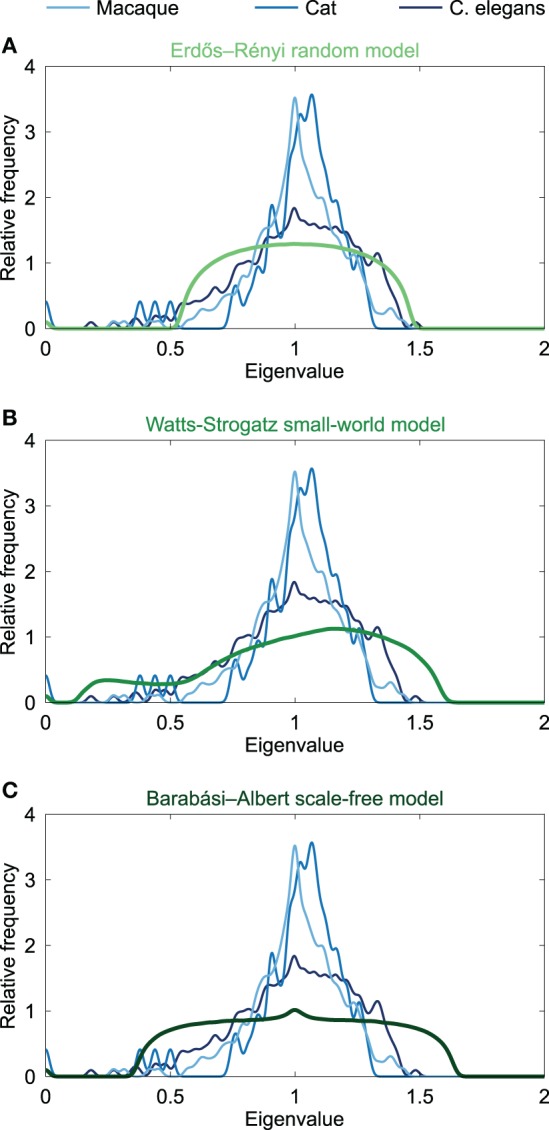
**Spectral plots of model networks**. Figure shows the Laplacian spectra of **(A)** the Erdös-Rényi random model, **(B)** the Watts-Strogatz small-world model, and **(C)** the Barabási-Albert scale-free model. The model spectra were averaged over a set of 1000 generated networks. All model networks had 274 nodes and densities close to 6% comparable to the C. elegans. The spectra of the neural networks of the macaque, cat and C. elegans are plotted in the background, and were found to be dissimilar from the three model spectra, suggesting a different global topological organization.

Given that similar spectra reflect similar network structures, it has been noted that the distribution of Laplacian eigenvalues can be used for classification of networks (Ipsen and Mikhailov, 2001; Vukadinović et al., 2002; Banerjee and Jost, 2008b; Cetinkaya et al., 2012). Spectral similarity was examined by visual inspection of the characteristics of the spectral plots and quantified by computing the average Euclidean distance between spectra. Comparing the Laplacian spectra of the neural networks to the spectra of conceptual Erdös–Rényi (ER) random networks (Erdös and Rényi, 1959), the Watts-Strogatz (WS) model for small-world networks (Watts and Strogatz, 1998) and Barabási-Albert (BA) model for scale-free networks (Barabási and Albert, 1999) (see Figure 2) revealed distinct dissimilarities. ER random networks showed an oval shaped Laplacian spectra, which were, except for a small bump at λ = 0 reflecting the number of connected components of the network, completely symmetric around one. WS small-world networks showed an asymmetric spectrum, with similarities to both the spectrum of a regular ring lattice and a random network (Banerjee and Jost, 2008b). BA scale-free networks showed symmetric spectra similar to that of the random network, but with a small peak around one. ER random and BA scale-free networks showed a maximum around one, but did not show the characteristic skewed distribution that was observed in the neural networks. In contrast, the spectra of the WS small-world networks showed a skewed distribution, but these spectra showed a maximum around 1.2 instead of having a peak around one, as compared to the neural spectra. In addition, the spectra of the WS small-world networks did not reveal scattered first eigenvalues as seen in the neural spectra. Confirming visual observation, the average Euclidean distance (see Table 1) between the Laplacian spectra of the macaque and cat connectomes revealed to be relatively small, indicating a high level of overlap between the anatomical communication infrastructure of the macaque and cat brain. Comparing across scales, the spectra of the C. elegans connectome and the cat and macaque brain networks were found to be more distant, suggesting relative differences between the spectra (Table 1). It appears that this distance results from differences in the density of the eigenvalues close to one, as the Laplacian spectra of the cat and macaque network showed a stronger peak around one than the Laplacian spectrum of the C. elegans connectome. In all, the spectra of the neural networks and model networks were found to be distant, suggesting that the neural networks were distinct from the conceptual ER random, WS small-world or BA scale-free models. These findings suggest a certain level of uniqueness of neural networks in comparison to these characteristic model networks in terms of global topological organization.

**Table 1:** **Spectral distances**.

	**Cat**	**C. elegans**	**ER**	**WS**	**BA**	**Food web**	**Email**	**Power grid**	**Football**
Macaque	0.0667	0.1249	0.2004 (0.0040)	0.3030 (0.0093)	0.3473 (0.0124)	0.0777	0.2175	0.4964	0.1409
Cat	–	0.1776	0.2610 (0.0042)	0.3310 (0.0088)	0.3835 (0.0119)	0.0866	0.2491	0.4392	0.1716
C. elegans		–	0.1034 (0.0039)	0.1761 (0.0078)	0.2336 (0.0129)	0.2181	0.1402	0.4797	0.0849
ER			–	0.2198	0.2143	0.2840 (0.0034)	0.6632 (0.0022)	0.5229 (0.0016)	0.1343 (0.0029)
WS				–	0.1654	0.3726 (0.0083)	0.8226 (0.0101)	0.3960 (0.0071)	0.1488 (0.0100)
BA					–	0.4092 (0.0104)	0.8359 (0.0047)	0.4850 (0.0058)	0.2672 (0.0132)
Food web						–	0.1124	0.4761	0.2191
Email							–	0.4057	0.1620
Power grid								–	0.4055

*Table summarizes the spectral distances (computed as the average Euclidean distance between the points of the two spectra) between the three neural networks (i.e., macaque, cat, C. elegans), the three model networks (ER: Erdös-Rényi random model, WS: Watts-Strogatz small-world model and BA: Barabási-Albert scale-free model) and 4 empirical networks. Values indicate mean distances and standard deviations*.

#### Comparison of neural networks to empirical networks

**Figure 3:**
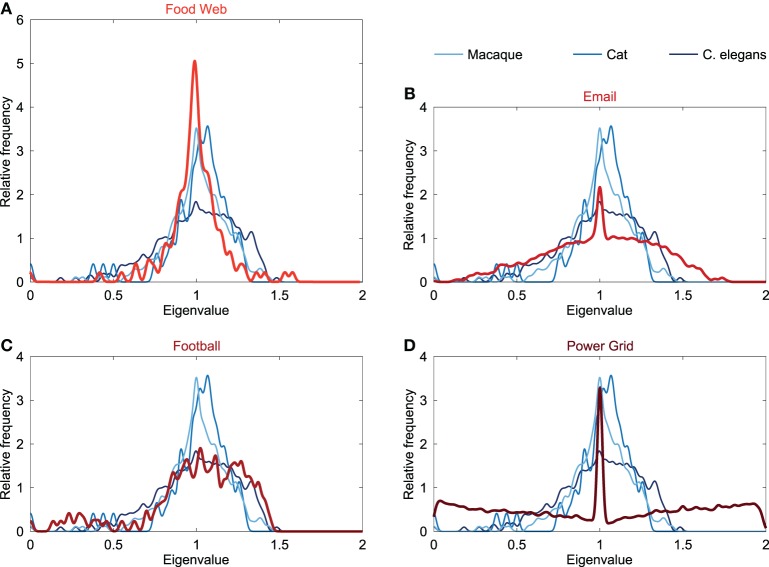
**Spectral plots of empirical networks**. Figure shows the Laplacian spectra of **(A)** a food web (121 components, 23.9% edge density), **(B)** email communication network (1133, 0.85%), **(C)** football games network (115, 9.35%), and **(D)** power grid (4941, 0.054%). The neural networks of the macaque, cat and C. elegans are plotted in the background and show only little similarity with the spectrum of the email and power grid network. The spectra of the food web and football network showed overlap with the neural spectra. The spectra of the food web and football network differed in the height of their peak around one, resulting the food web to be spectrally closest to the macaque and cat networks, whereas the football network was found to be the closest to the C. elegans network (see also Table 1).

In addition to the comparison of the neural spectra to the spectra of conceptual models, the Laplacian spectra of neural networks were also compared to an ensemble of empirical networks. Empirical networks included the Food web in Florida during wet season (referred to as “Food web”), the email interchange network at the university of Rovira i Virgili (Email network), a network of American football games between colleges in the United States (Football network) and the power grid of the Western States of the United States (Power grid). Spectra of these networks are plotted in Figure 3.

The spectrum of the power grid and email network (Figure 3) both showed a maximum around one, but did not display an asymmetrically skewed spectrum or scattered first eigenvalues, opposed to the spectra of the neural networks. Furthermore, the power grid and email network showed a large spectral distance to the neural networks (Table 1), suggesting differences in global topological organization between these two networks and the neural networks.

The food web showed a spectrum with a maximum at one and with the first eigenvalues scattered (Figure 3), overlapping with the characteristics of the neural spectra. However, in contrast to neural spectra, the spectrum of the food web showed to be highly symmetric, with a weaker community structure compared to the neural networks (see below). The high peak around one in the spectrum of the food web was similar to the peak observed in the cat and macaque. In terms of spectral distances, the food web was found to be closer to the macaque and cat network than to the C. elegans network (Table 1). This suggests that the food web shares, to some extent, structural properties with neural networks, in particular the organization of macroscopic neural networks.

The football network showed a similar skewed asymmetric spectrum as reported for the neural networks (Figure 3). Furthermore, the first eigenvalues were scattered likewise to the neural spectra, but higher levels of eigenvalues below 0.5 were observed. The football spectrum displayed a maximum at one and, similar to the spectrum of the C. elegans, there was only a modest peak observed around this maximum (Figure 3). These resemblances were also found when evaluating the spectral distances, showing that the football spectrum was spectrally closest to the C. elegans network, but distant from the macaque and cat neural network. This indicates a relatively higher similarity in global topological organization between the football network and the C. elegans connectome, than with the macaque and cat networks.

### Spectrum properties: smallest eigenvalues

#### General description

**Figure 4:**
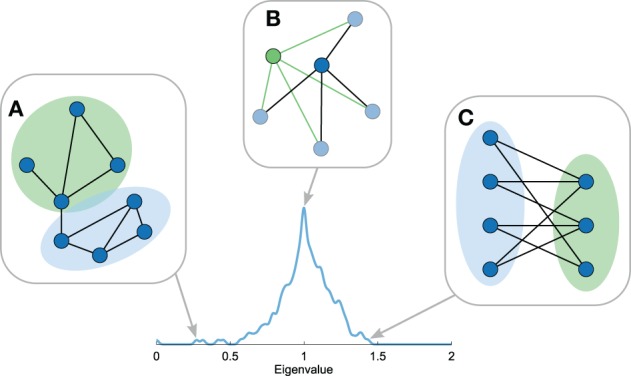
**Topological properties reflected in the Laplacian spectrum**. Figure shows several properties of the Laplacian spectra. **(A)** The first eigenvalues reflect community structure with smaller first eigenvalues indicating stronger community structures. **(B)** Motif manipulations, i.e., motif addition or duplication, result in explicit eigenvalues in the spectrum. Hence, recursive manipulations can result in eigenvalues with high multiplicities, giving rise to peaks in the Laplacian spectrum. **(C)** The largest eigenvalue reflects the level of bipartiteness of the most bipartite subpart of the network, which is closely related to the number of odd cyclic motifs in the network.

The smallest eigenvalues of the Laplacian spectrum -meaning the first few eigenvalues of the labeled spectrum with 0 = λ_1_ ≤… ≤ λ_*n*_ ≤ 2 – include information on the community structure of a network (Figure 4A) (Shi and Malik, 2000; Donetti and Munoz, 2005; Fortunato, 2010; Shen and Cheng, 2010). Each eigenvector *v*_*i*_ describes a unique bisection of the network by assigning a positive or negative value to each node (the eigenvector components) and the associated eigenvalue λ_*i*_ describes the inverse diffusion time of this division to the stationary state (Cheng and Shen, 2010). As such, smaller eigenvalues indicate longer diffusion times, revealing a large proportion of intramodule connections and a low number of intermodule connections. The smallest non-zero eigenvalue λ_2_ (in connected networks) thus provides information on the best possible cut of the network into two modules (Chung, 1996). The divisions of all eigenvectors up to *v_i_* can subsequently be combined to separate the nodes of the network into *i* communities, where a possible number of communities for the optimal division might be suggested by the largest eigengap (λ_*i* + 1_ − λ_*i*_) (Shi and Malik, 2000; Cheng and Shen, 2010). In all, the number of small eigenvalues, their values, and eigengaps reflect aspects of the community structure of the network.

#### Macaque

The smallest non-zero eigenvalue of the macaque dataset was found to be λ_2_ = 0.2718, which is significantly lower compared to the randomized reference networks (see methods, 1000 random networks were examined, with an average λ_2_ of 0.61 ± 0.06 [mean ± standard deviation], *p* < 0.001, permutation testing). This finding indicates a good cut of the network into two parts and thereby a relative high level of community structure. This result was endorsed by λ_6_− λ_5_ = 0.1153 being the largest eigengap, indicating that an optimal division of the network is suggested to be into 5 communities.

#### Cat

For the cat dataset, the smallest eigenvalue revealed to be λ_2_ = 0.3782, which was again significantly lower compared to matched random networks, having an average λ_2_ = 0.6937 ± 0.0134 (*p* < 0.001). Additionally, λ_5_ − λ_4_ = 0.2512 was the largest eigengap, suggesting an optimal division of the network into four communities.

#### C. elegans

For the C. elegans network, the smallest eigenvalue λ_2_ = 0.1818 was also found to be significantly lower relative to randomized networks (λ_2_ = 0.5438 ± 0.0142, *p* < 0.001). Although various small eigenvalues showed relatively large eigengaps, demonstrating the existence of a number of stable communities, the eigengap λ_2_− λ_1_ revealed to be the largest, indicating that there is no clear optimal network division of the neural network map of the C. elegans. This showed that although there is a certain level of modular structure within the C. elegans connectome dataset, the community structure is relatively weak.

### Spectrum properties: largest eigenvalue

#### General description

The largest eigenvalue of the Laplacian spectrum provides information on the level of “bipartiteness” of (subparts of) a network (Figure 4C) (Bauer and Jost, 2012). A (sub)network is said to be (fully) bipartite if its nodes can be divided into two groups in such a way that nodes within the same group are not connected. As a result, bipartite networks lack cycles with an odd number of nodes. A division of nodes on basis of the positive and negative values in the largest eigenvector indicates the most bipartite configuration of the network. The largest eigenvalue λ_*n*_ reflects the level of bipartiteness of this configuration. Considering that it is possible that only a subset of the network is explicitly positive or negative in the largest eigenvector, λ_*n*_ may not be a metric reflecting global properties of a network *per se*.

#### Macaque

The largest eigenvalue λ_*n*_ of the macaque was reported to be equal to 1.4341, not significantly higher than observed in a set of comparable networks with a randomized structure (λ_*n*_ = 1.3927 ± 0.0558 and *p* = 0.2291). Additional visual inspection of the eigenvector linked to the largest eigenvalue, presented with respect to communities as identified in Harriger et al. (2012), showed that this eigenvector was localized on two of the five structural modules, identified to include frontal/orbitofrontal and temporal regions (Harriger et al., 2012).

#### Cat

The largest eigenvalue in the cat dataset was found to be λ_*n*_ = 1.2875. For comparable random networks, the largest eigenvalue was on average 1.3319 ± 0.0119, indicating that the level of bipartiteness of the cat cortex was significantly lower (*p* < 0.001) as compared to that in random networks. Visual inspection of the associated eigenvector showed that this eigenvalue represented the global network, suggesting an overall weaker bipartiteness in the network of the cat cortex, indicating more odd cyclic motifs (i.e., non-reducible cycles with an odd number of nodes) in the cat cortex compared to random networks.

#### C. elegans

The largest eigenvalue of the C. elegans showed to be λ_*n*_ = 1.4827, which was higher than in comparable random networks (λ_*n*_ = 1.4613 ± 0.0136), although this effect was only marginal and present at a trend-level (*p* = 0.0574). Additional visual inspection showed that the eigenvector associated with this eigenvalue was restricted to one of the four communities identified (Newman, 2006). This community included mostly ventral cord motor neurons, indicating that this class constitutes a relative high bipartite community.

### Spectrum properties: motif manipulations

#### General description

Repeated addition and duplication of nodes and motifs in the development of a network has been shown to leave traces in a network's Laplacian spectrum (Banerjee and Jost, 2008a, 2009). For example, node duplication, in which one new node is introduced to the network that has the same connectivity pattern as the duplicated node resulting in two nodes with an identical connectivity profile, has been shown to result in an increase in the eigenvalue λ = 1 of the spectrum (Figure 4B) (Banerjee and Jost, 2008a). Hence, recursive node duplication results in a characteristic high multiplicity of the eigenvalue λ = 1 in the spectrum. Duplication of an edge motif, i.e., duplication of two connected nodes *j*_1_ and *j*_2_ and their connections, has been noted to produce eigenvalues symmetric around 1 with eigenvalues $\lambda_{\pm }=1\pm (1/\sqrt{d_{j1}d_{j2}}$), with *d*_j_ being the degree of node *j*. Also addition of motifs to the network generates specific eigenvalues, with for example the inclusion of a new triangle motif to a network resulting in the addition of an eigenvalue λ = 1.5 to the spectrum (Banerjee and Jost, 2008a). In general, motif joining or duplication produces specific exact eigenvalues and subsequent repetition of these processes result in characteristic high eigenvalue multiplicities, visible as peaks in the Laplacian spectrum. Hence, the presence of eigenvalues with high multiplicities (e.g., a high peak at λ = 1) or eigenvalues at equal distances to 1 may provide an indication of local organizations resulting from recursive motif manipulations.

#### Macaque, cat and C. elegans

The spectra of all three neural networks showed a peak around one, suggesting traces of node duplication. Additionally, the observed peaks around one revealed to be rather symmetric, indicating traces of edge duplication in the networks. Comparison between the neural spectra showed that the peak around one and its symmetry was most clearly seen in the spectra of the macaque and cat network and to a lesser extent in the C. elegans neural system. This might indicate higher levels of duplication in the networks of the macaque and cat compared to that of the C. elegans. The spectra showed no other clear peaks indicative of recurrent addition of motifs.

### Robustness analysis

**Figure 5:**
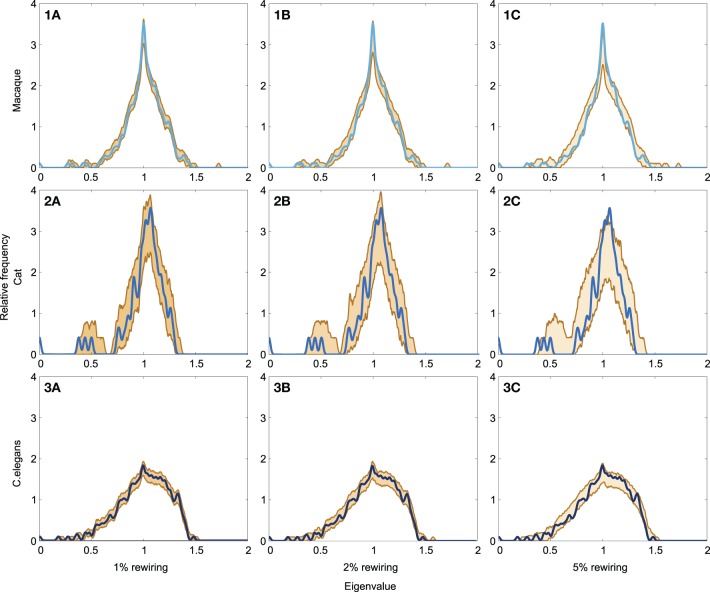
**Robustness of neural network spectra to edge rewiring. (A1)** Spectrum of the macaque with 1% of the edges rewired, **(A2)** spectrum plotted with 2% rewiring, and **(A3)** spectrum plotted with 5% rewiring. (**B1–3)** show the spectra of the cat network with added noise. (**C1–3)** show the spectra of the C. elegans network with added noise. The figure shows the lower and upper boundary of the 1000 iterations for each eigenvalue. Across all three rewiring levels, the figures show a high level of consistency between the spectra across original and noise including networks. The cat network (**C1–3**) appears the most affected by the rewiring processes, which is likely a consequence of its relatively small network size. In general, the spectra of the “noise including networks” show large overlap with the original spectra, indicative of the spectra to be robust to small modifications to the datasets.

The level of robustness of the obtained spectra in relation to (small) amounts of noise, reflecting modifications to the original connectome datasets, was examined through means of a robustness analysis. Noise was simulated by means of randomly rewiring a (small) proportion of the edges in the networks (ranging from 1 to 5%, preserving network size, density and degree distribution) over a series of iterations (1000). For each of the obtained “noise including networks” the spectra was computed and compared to the original network. The lower and upper boundary of spectra of the 1000 “noise including networks” are shown in Figure 5, together with the original networks, for each of the three examined neural networks (panel 1A–C: macaque, panel 2A–C: cat, panel 3A–C: C. elegans). Overall, large overlap (as expressed by small spectral distances) was found between the original and noise including networks, for all three neural networks (see Table 2). As expected, with the inclusion of increasing levels of random noise (from 1 to 5%), the spectra of the noise including neural networks started to reveal slightly more random properties, including higher smallest eigenvalues and a less steep peak around one.

**Table 2:** **Robustness of neural network spectra to edge rewiring in spectral distance**.

	**1% rewired**	**2% rewired**	**5% rewired**
Macaque	0.0124 (0.0022)	0.0189 (0.0030)	0.0352 (0.0044)
Cat	0.0185 (0.0050)	0.0281 (0.0076)	0.0575 (0.0112)
C. elegans	0.0159 (0.0023)	0.0217 (0.0023)	0.0317 (0.0028)

*Table shows the spectral distances between the neural networks (macaque, cat and C. elegans) and the noise including neural networks in which a small proportion (1, 2, and 5%) of the edges in the original networks were randomly rewired. Values indicate mean distances and standard deviations*.

## Discussion

The main finding of this paper was a common underlying structural organization of neural networks across species. Examining and comparing the Laplacian spectrum of the macroscopic or microscopic neural network maps of the macaque, cat and C. elegans connectome, it was found that the neural spectra showed mutual overlap on several characteristics. Furthermore, no clear overlap between spectra of the macaque, cat and C. elegans and the spectra of strict, conceptual ER random, WS small-world or BA scale-free models could be observed (Figure 2), nor between the neural spectra and the spectra of an email network and a power grid network (Figure 3). As such, our findings indicate a relatively unique shape of the spectra of neural networks, suggesting that neural networks may form, to some extent, a type of special network class.

Examination of the small eigenvalues in the neural spectra revealed a community structure in all of the three networks, consistent with earlier observations showing community structure in neural networks (Hilgetag et al., 2000; Arenas et al., 2008). Furthermore, the largest eigenvalue reflecting the level of bipartiteness of a network revealed to be significantly smaller in the cat dataset as compared to random networks, suggesting a lower level of bipartiteness in the cat network compared to random networks. As bipartiteness is strongly related to the presence of odd cyclic motifs, this would agree with commonly observed global small-world properties of neural networks, predicting more triangle motifs (Bassett and Bullmore, 2006; Hagmann et al., 2008; van den Heuvel et al., 2008b; Bullmore and Sporns, 2009). However, in the dataset of the macaque and C. elegans this effect was less clear, with the largest eigenvalue indicating random levels of bipartiteness. This may suggest that these neural networks, or at least one community in these neural networks, show similar odd cycle levels as seen in random wiring. This was unexpected, since the small-world property of brain networks implies a high (normalized) clustering coefficient, indicating the existence of more triangle motifs than in random networks. An explanation for this observation would be the absence of non-reducible odd cyclic motifs with five nodes or more. The symmetric peak around one observed in all three neural spectra suggested traces of node and edge duplication in the neural networks. The macaque and cat spectra showed more evident peaks around one compared to the spectrum of the microscopic C. elegans network, suggesting higher levels of duplication in the macroscopic networks of the macaque and cat.

Considering the relative strong overlap of the three types of examined neural networks, and the relative high distance between the spectra of the three neural networks and the spectra of the simple model ER random, WS small-world and BA scale-free networks tends to suggest that the rich architecture of neural networks cannot be simply explained by one of these simple network models. However, this does not imply that neural networks do not show small-world (i.e., high clustering and short paths) or scale-free properties (i.e., heavy tailed degree distributions), but merely suggests that the rich architecture of neural networks cannot be explained by one of these simple network models alone. WS small-world and BA scale-free networks only represent an example class of networks that display small-world and scale-free properties. Indeed, several network studies have shown that neural networks of the human brain deviate from the simple BA scale-free or WS small-world network model. For example, studies have suggested that low resolution networks tend to display a truncated power-law degree distribution rather than a power-law with an exponent around 2.9 as seen in BA scale-free networks (Barabási and Albert, 1999; Achard et al., 2006). Furthermore, studies have suggested that neural networks display properties such as a rich modularity structure (Salvador et al., 2005; van den Heuvel et al., 2008a; Rubinov and Sporns, 2011) and high levels of intermodular connectivity (de Reus and van den Heuvel, 2013; van den Heuvel and Sporns, 2013b), properties that cannot be expected from WS small-world networks derived from rewiring a small number of edges from an otherwise lattice architecture. The observed relatively unique shape of the neural spectra of the examined neural networks does suggest that these neural networks exhibit a rich repertoire of network attributes, together forming an underlying architectural organization that cannot be properly described by one simple model network class (e.g., ER random, WS small-world or BA scale-free).

The used methodology, as well as the spatial resolution at which connectivity datasets are acquired, is likely to have an effect on the reported network structure and thus their spectrum (Bullmore and Sporns, 2009). In this study, we examined the spectra of the anatomical networks of the cat, macaque and C. elegans. These neural networks are commonly referred to in the literature and widely considered as reference connectome datasets for these species (Hilgetag et al., 2000; Kötter, 2004; Sporns and Kötter, 2004; Kaiser, 2011; Zamora-López et al., 2011; de Reus and van den Heuvel, 2013). However, general caution is needed when comparing network attributes across species, and, although the three examined networks are interpreted as representatives of the “class of neural networks,” we note that it remains unclear whether, and if so to what extent, our findings can be generalized to the neural networks of other species and to neural networks of other modalities. A few comments are in place. First, connectome research is a rapidly growing field and the connectivity data as used in this paper is likely to be complemented by more detailed data in the near future, which may result in (small) modifications to the connectivity matrices. To understand the robustness of the spectrum to such modifications and noise in general, the effects of modest modifications to the neural network maps was simulated by randomly rewiring a small proportion (1–5%) of the edges in the networks. Spectra of these noise including neural networks were found to show large overlap with the original networks, illustrating a high level of robustness to small variation in overall wiring layout (Figure 5). These findings suggest that small changes to the neural networks examined in this study have only limited effect on the shown neural spectra. However, second, the neural datasets examined in this study describe anatomical connectivity, incorporating results from tracer studies (macaque and cat) and studies using electron microscopy (C. elegans). It remains unclear whether the spectra of these three neural networks can be generalized to the spectra of anatomical networks of other species, including human. Third, the examined spectra might differ from spectra of structural brain networks obtained by other techniques to reconstruct anatomical connectivity, such as Diffusion MR Imaging (Hagmann et al., 2008; Iturria-Medina et al., 2008; van den Heuvel et al., 2010). Furthermore, in our study we examined the spectra of anatomical networks, but these spectra might differ from spectra of functional brain networks derived from functional MRI data. Studies have suggested that the architecture of structural and functional networks are related, but their relationship is complex (Hagmann et al., 2008; Bullmore and Sporns, 2009; van den Heuvel and Sporns, 2013a). This is likely to result in differences in the spectra of functional networks as compared to the spectra of anatomical networks. Future studies examining the spectra of networks derived from diffusion imaging and resting-state fMRI, in particular of human data, are needed to examine to what extent our findings can be generalized across neural networks.

In context of our study, it might be worth to note that some of the spectral differences observed between networks may be accounted for by external factors not directly related to the network's topological features. First, in networks of smaller size, such as that of the cat, small variations in the eigenvalues have relatively great impact on the spectrum. Hence, in these networks, small spikes in the spectra should be interpret with caution, and are likely not to reflect global network properties. Indeed, simulation of noise showed to have the largest effect on the cat dataset (Figure 5). Second, the size and density of a network, describing the number of nodes and edges representing the network, might have a strong influence on the distribution of the eigenvalues (Chung et al., 2003). In random networks, higher density is associated with a larger λ_2_ and smaller λ_*n*_ and this trend is consistent with reported differences in λ_2_ and λ_*n*_ between the macaque, cat and C. elegans spectra. Third, in the analysis all networks were regarded as binary and undirected, which made the connectivity data less accurate. An exploratory analysis including information on the directionality and weighting of the connections in the networks was included in the Supplementary Material. In this supplementary analysis, the method proposed by Chung (2005) was used to ensure real eigenvalues. However, this transformation does not exploit the directionality of the connectivity data to the full extent possible, and future studies examining the Laplacian spectrum of directed networks in more detail (including detailed information on complex eigenvalues) would be of high interest.

The Laplacian spectra of the food web and football network showed resemblances with the spectra of the neural networks, with the food web spectrum spectrally closest to the spectra of the macroscopic macaque and cat network and the spectrum of the football network closest to the spectrum of the microscopic C. elegans network. This dissociation in spectral distance might indicate differences in the structural organization of the neural networks. The football network has a geographically constrained community structure, with a high number of matches between teams from the same conference and sparse interconference matches that have a strong preference for geographically close teams (Girvan and Newman, 2002). Conversely, the food web is the result of a long interplay between the introduction of new species resulting from differentiation and immigration, and the extinction of species on basis of their fitness (Drossel and McKane, 2002). Hence, the spectral similarities between the neural networks and the food web and football network possibly suggest that neural networks on the microscopic level share organizational properties related to a spatially driven community structure, whereas as macroscopic interactions might be shaped by a long developmental trajectory influenced by evolutionary constraints. Such growth constrains would be in agreement with a proposed balance in brain architecture between minimizing wiring cost and advantageous topological properties such as efficiency or robustness (Bullmore and Sporns, 2012; Collin et al., 2013; van den Heuvel and Sporns, 2013a).

Extending previous findings on the architectural organization of neural networks, our findings show a high level of similarity of the Laplacian spectra across the cat, macaque and C. elegans neural networks, indicative of a relatively high level of consistency and conservation of overall network structure of neural systems. Our findings suggest that neural networks display an architecture that includes a rich repertoire of network attributes, forming a relative unique network class that is distinct from simple conceptual WS small-world and BA scale-free models, as well as from several empirical networks.

### Conflict of interest statement

The authors declare that the research was conducted in the absence of any commercial or financial relationships that could be construed as a potential conflict of interest.
