# Supplementary material for: The Laplacian spectrum of neural networks
Source: Front Comput Neurosci. 2014 Jan 13;7:189. doi: 10.3389/fncom.2013.00189 (PMC3888935; doi:10.3389/fncom.2013.00189)
Supplement: Supplementary file 1 [file Data_Sheet_1.PDF]

Supplementary Material

The directed Laplacian spectrum

This paper investigated the anatomical neural networks of the macaque, cat and Caenorhabditis elegans using the well-documented undirected and unweighted normalized Laplacian spectrum, reflecting the elementary topological properties of these networks. Facilitating further analysis, taking into account information on the directionality and connection strength of synapses and white matter tracts, two (normalized) directed Laplacians have been suggested (Chung, 2005; Bauer, 2012). The spectra of both directed Laplacians agree for undirected networks, however when directed connections are present, the directed Laplacian by Bauer shows complex eigenvalues, whereas the eigenvalues of the directed Laplacian by Chung remain real and between 0 and 2. Although the directed Laplacian by Bauer is likely to incorporate more information in its complex spectrum, we restrict ourselves to the spectrum of the directed Laplacian by Chung, as the interpretation of its spectrum is more comparable to that of the undirected Laplacian.

The directed Laplacian (Chung, 2005) is defined as

$$L = I - \frac{\Phi^{1/2} P \Phi^{-1/2} + \Phi^{-1/2} P^{*} \Phi^{1/2}}{2}$$

where $P=D^{-1}W$, with W being the weighted connectivity matrix, D a weighted diagonal matrix with the strength of each node on the diagonal, P^* the conjugated transpose of P and Φ being the diagonal matrix with the left eigenvector φ of P associated with eigenvalue one on the diagonal (normalized such that $\sum \varphi = 1$). The directed Laplacian is applicable to directed networks that have only positively weighted connections and that are strongly connected, i.e. for any pair of distinct vertices *i* and *j* there exists a path from *i* to *j* and a path from *j* to *i* (Chung, 2005).

The eigenvalue interpretations of the undirected Laplacian cannot directly be generalized to the spectrum of the directed Laplacian. Until now, it has only been shown that the small eigenvalues of the directed Laplacian reflect network parts that are loosely connected (Chung, 2005). However, the eigenvalues of the directed Laplacian and undirected Laplacian coincide on undirected networks, suggesting similarities between the structural properties of the eigenvalues to a certain extent.

The original connectivity datasets of the three neural networks were all directed and the datasets of the cat and C. elegans were also weighted. Comparing the weighted directed and binary undirected spectra of the neural networks showed greater spread of the extreme eigenvalues (see Supplemental Figure 1). Greater spread of the spectra is reflected in lower smallest eigenvalues and higher largest highest eigenvalues: for the network of the macaque was found λ_2=0.1992 and λ_n=1.5686, for the network of the cat was reported λ_2=0.2334 and λ_n=1.3821 and the C. elegans connectome showed λ_2=0.0864 and λ_n=1.7548. Furthermore, the directed Laplacian spectra of the macaque and C. elegans showed a bigger peak around one than the undirected Laplacian spectra, while the directed spectrum of the cat network reported a smaller peak around one.

The finding that the first eigenvalues were smaller in the directed spectra compared to the first eigenvalues in the undirected spectra, suggests a stronger community structure in the directed networks. However, further research is needed, as such effects might also be influenced by the lower density of the directed networks. Alternatively, differences in spectra might result from differences in the hierarchical structure of the networks. In all, it stays unclear to what extent the eigenvalues of both Laplacians are directly comparable, but our preliminary

findings suggest that the characteristics found in the undirected spectra of the neural networks are also largely present in the directed spectra.

References

Bauer, F. (2012). Normalized graph Laplacians for directed graphs. *Linear Algebra and its Applications* 436, 4193–4222. doi:10.1016/j.laa.2012.01.020

Chung, F. R. K. (2005). Laplacians and the Cheeger Inequality for Directed Graphs. *Annals of Combinatorics* 9, 1–19. doi:10.1007/s00026-005-0237-z

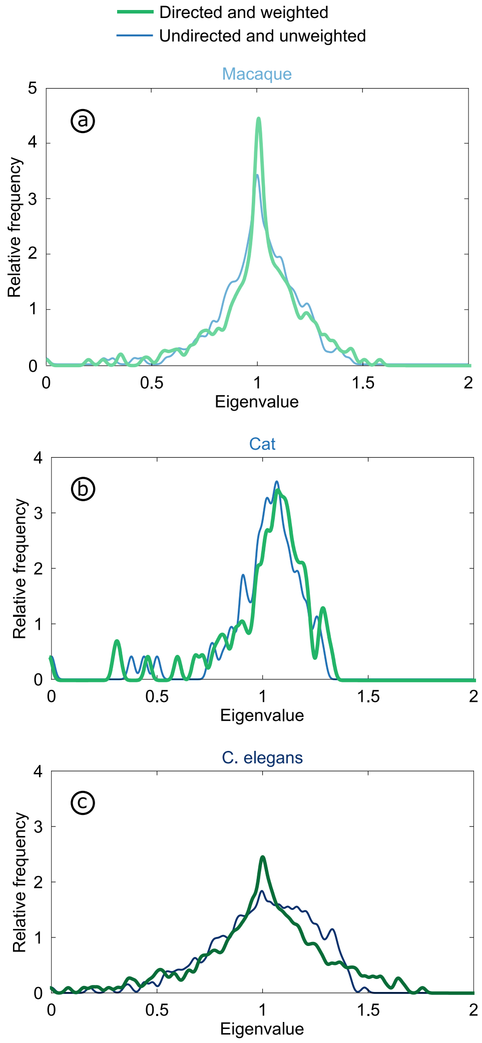

Supplemental Figure 1. The weighed and directed Laplacian spectra of the neural networks. (A) Spectrum of the unweighted directed macaque network (242 cortical regions, 7.0% sparsity). (B) Spectrum of the weighted directed cat network (65 cortical brain areas, 27.4%). (C) Spectrum of the weighted directed C. elegans network (274 neurons, 3.95%). The unweighted undirected Laplacian spectra of the neural networks, as discussed in the main text, are plotted in the background. Comparison between the directed and undirected Laplacian suggests that the directed spectra are more spread out, but that the global shape of the spectra is comparable.
